# Genome collection of *Shewanella* spp. isolated from spoiled lamb

**DOI:** 10.3389/fmicb.2022.976152

**Published:** 2022-09-27

**Authors:** Nikola Palevich, Faith P. Palevich, Amanda Gardner, Gale Brightwell, John Mills

**Affiliations:** AgResearch Ltd., Grasslands Research Centre, Palmerston North, New Zealand

**Keywords:** *Shewanella*, genome sequence, meat spoilage, bacteria, stress adaptation

## Abstract

The diversity of the genus *Shewanella* and their roles across a variety of ecological niches is largely unknown highlighting the phylogenetic diversity of these bacteria. From a food safety perspective, *Shewanella* species have been recognized as causative spoilage agents of vacuum-packed meat products. However, the genetic basis and metabolic pathways for the spoilage mechanism are yet to be explored due to the unavailability of relevant *Shewanella* strains and genomic resources. In this study, whole-genome sequencing of 32 *Shewanella* strains isolated from vacuum-packaged refrigerated spoiled lamb was performed to examine their roles in meat spoilage. Phylogenomic reconstruction revealed their genomic diversity with 28 *Shewanella* spp. strains belonging to the same putative novel species, two *Shewanella glacialipiscicola* strains (SM77 and SM91), *Shewanella xiamenensis* NZRM825, and *Shewanella putrefaciens* DSM 50426 (ATCC 8072) isolated from butter. Genome-wide clustering of orthologous gene families revealed functional groupings within the major *Shewanella* cluster but also considerable plasticity across the different species. Pan-genome analysis revealed conserved occurrence of spoilage genes associated with sulfur and putrescine metabolism, while the complete set of trimethylamine metabolism genes was observed in only *Shewanella* sp. SM74, *S. glacialipiscicola* SM77 and SM91 strains. Through comparative genomics, some variations were also identified pertaining to genes associated with adaptation to environmental cues such as temperature, osmotic, salt, oxidative, antimicrobial peptide, and drug resistance stresses. Here we provide a reference collection of draft *Shewanella* genomes for subsequent species descriptions and future investigations into the molecular spoilage mechanisms for further applications in the meat industry.

## Introduction

The genus *Shewanella* was first validly published in 1985 (MacDonell and Colwell, 1985) and was included within the family *Shewanellaceae* in 2004 (Ivanova et al., 2004), having been moved from its previous position within the *Alteromonadaceae*. A total of 76 species are currently recognized as being validly published with correct names within this taxonomic group (Parte et al., 2020). The genus comprises species that are ubiquitous in aquatic environments, being found in both hot and cold climates, marine and fresh waters, deep-sea sediments, and oil field wastes (Nealson and Scott, 2006). Several *Shewanella* species have been associated with emerging opportunistic human infections (Holt et al., 2005; Janda and Abbott, 2014; Fang et al., 2017; Martín-Rodríguez and Meier-Kolthoff, 2022), while others, notably *Shewanella putrefaciens* and *Shewanella baltica*, are implicated in the spoilage of high pH meat products and seafood (Gill and Newton, 1979; Gram et al., 1987; Vogel et al., 2005).

The pH of beef, lamb, and venison can range from 5.3 to 6.9 depending on the species, muscle type, slaughter season, and other variables; however, meat presenting desirable eating attributes typically has a pH < 5.8, whereas meat with pH > 5.8 (other than lamb shoulder and shank) has inferior qualities such as darker color and shorter shelf life (Reis and Rosenvold, 2014). On high pH meats (pH > 6.0), *S. putrefaciens* has been shown to cause spoilage both aerobically and anaerobically, utilizing the sulfur-rich amino acids serine and cysteine even when glucose is abundant, producing organic sulfides that contribute to off odors and flavors (Gill, 1986). Anaerobically in vacuum packs, while not fermentative, these sulfides are utilized as alternate terminal electron acceptors rather than oxygen to maintain respiratory metabolism (Gill and Newton, 1979); the abundant hydrogen sulfide (H_2_S) produced causes green sulfmyoglobin discoloration at the meat surface as well as degradation of the odor and flavor (Devine and Dikeman, 2014). On fish, *Shewanella* become dominant organisms during storage, producing deleterious odors due to trimethylamine oxide (TMAO) reduction to trimethylamine, and production of H_2_S (Nealson and Scott, 2006).

*Shewanella putrefaciens* DSM 50426 (ATCC 8072) isolated from butter and all additional 31 *Shewanella* spp. strains isolated from vacuum-packaged refrigerated spoiled lamb are psychotropic, facultative anaerobic, Gram-negative gammaproteobacteria that inhabit diverse environments and their phylogenetic relationships are largely uncharacterized (Lemaire et al., 2020). By studying the *Shewanella* genomes, we aim to determine their biochemical mechanisms and spoilage pathways that enable these species to metabolize sulfur-containing amino acids or proteins to produce H_2_S resulting in a fishy odor that is characteristic of *Shewanella*-spoiled meat.

## Materials and methods

### Bacterial cultivation and growth conditions

*Shewanella putrefaciens* DSM 50426 (ATCC 8072) was acquired from the Leibniz Institute DSMZ-German Collection of Microorganisms and Cell Cultures, and *Shewanella xiamenensis* NZRM825 from the NZRM Culture Collection. The remaining 30 *Shewanella* spp. strains were isolated from vacuum-packaged lamb that had no pack distension, with meat discoloration (some green spots) and sweaty feet odor for some strains. Meat samples were added into Whirl-Pak bags with 50 ml 0.1% peptone diluent (Fort Richard Laboratories) and stomached (Seward, United Kingdom) for 2 min at full speed. The liquid suspension was transferred to 50 ml falcon tubes and centrifuged at 5,000 × *g* for 10 min. The supernatant was discarded, and the pellet resuspended in 10 ml 0.1% peptone diluent. The suspensions were diluted 10-fold in 0.1% peptone diluent and the 0.1 ml of the dilutions plated onto Peptone iron agar (Fort Richard Laboratories). Plates were incubated at 25°C for 2 days. Individual black colonies on the Peptone iron agar were streaked onto Columbia Sheep Blood agar (Fort Richard Laboratories) to obtain pure colonies. Isolate identity was checked by PCR using 16S rRNA universal primers pA (5′-AGA GTT TGA TCC TGG CTC AG-3′) and pH (5′-AAG GAG GTG ATC CAG CCG CA-3′; Edwards et al., 1989).

### Preparation of genomic DNA and whole-genome sequencing

A single colony from each isolate was used to inoculate 10 ml TSB and incubated at 25°C for 2 days. Cells were pelleted by centrifugation at 5,000 × *g* for 10 min. High-molecular-weight genomic DNA was extracted using a modified phenol-chloroform procedure (5). Bacterial strain identity was verified by 16S rRNA gene amplification and sequencing of genomic DNA was verified by automated Sanger sequencing of the 16S rRNA gene following PCR amplification from genomic DNA. Total DNA concentrations were determined using a NanoDrop® ND-1000 (Thermo Scientific Inc.) and a Qubit Fluorometer dsDNA BR Kit (Invitrogen, United States), in accordance with the manufacturer’s instructions. Genomic DNA integrity was verified by agarose gel electrophoresis and using a 2000 BioAnalyzer (Agilent, United States). DNA libraries were prepared using the Illumina Nextera method chemistry kit v1 according to the manufacturer’s protocol and samples were sequenced to the requested depths on the Illumina MiSeq 500 platform (2 × 250 bp) to generate paired-end reads.

### Genome assembly and annotation

Reads were examined with FastQC v0.11.5 (Andrews, 2010), trimmed with Trimmomatic v0.39 (Bolger et al., 2014), and assembled using the A5-miseq pipeline v20169825 with standard parameters (Coil et al., 2015). The *de novo* assemblies were annotated using NCBI Prokaryotic Genome Annotation Pipeline (PGAP) v5.2 (Tatusova et al., 2016) and Blast2GO suite (Götz et al., 2008). CheckM (Parks et al., 2015) was used to estimate genome completeness with default software settings and parameters were used throughout unless specified otherwise.

### Phylogenomic analyses

Taxonomic assignments of our strains were explored initially by pairwise comparison of their Average Nucleotide Identity (ANIb) and correlation indexes of their Tetra-nucleotide signatures (TETRA) using JSpeciesWS v3.8.2 (Richter et al., 2016). In addition, whole nucleotide sequences were uploaded to the Type (Strain) Genome Server (TYGS) for whole-genome-based taxonomic analysis (Meier-Kolthoff and Göker, 2019). For the phylogenomic inference, all pairwise comparisons among the set of genomes were conducted using Genome BLAST Distance Phylogeny (GBDP) and accurate intergenomic distances were inferred under the algorithm “trimming” and distance formula *d*_5_ (Meier-Kolthoff et al., 2013). One hundred distance replicates were calculated each, and digital DDH values and confidence intervals were calculated using the recommended settings of the Genome-to-Genome Distance Calculator (GGDC)1 v3.0^1^ under recommended settings. Pairwise digital DNA:DNA hybridization values (dDDH) were inferred accordingly with the resulting distance matrix subjected to clustering based on established thresholds for delineating species (DDH > 70%) and subspecies (DDH > 79%), respectively (Meier-Kolthoff et al., 2013). One hundred pseudo-bootstrap replicates were assessed under the same settings each. Finally, a balanced minimum evolution tree was inferred using FastME v2.1.4 with SPR postprocessing (Lefort et al., 2015).

### Gene family evolution

To determine homologs and investigate orthologous gene (OG) family groups, the annotated protein sequences for all *Shewanella* genomes were analyzed using to OrthoFinder v2.5.2 (Emms and Kelly, 2019) with default settings. The resulting ortho-group counts were imported into R v4.1.1^2^ and the top 20 most common were displayed using UpSetR v1.4.0 (Conway et al., 2017).

## Results and discussion

### Genome characteristics and properties

The *Shewanella* spp. genomes assemblies ranged between 32 and 193 contigs, 4.1 to 5.5 Mbp in size, N_50_ contigs between 66,739 and 625,775 bp, G + C content of 44.1–46.3%, and a mean genome coverage between 45- and 87-fold (Table 1). Overall, the total numbers of annotated protein-coding genes (PCGs) ranged from 3,856 to 5,240, with 108–131 RNA genes consisting of 11–19 rRNAs and 96–112 tRNAs identified. The genome sequence features and data (NCBI SRA, GenBank, and Biosample database accessions) of the *Shewanella* spp. strains are summarized in Table 1.

**Table 1:** General genome features of *Shewanella* spp. strains.

Species	Strain	Size (bp)	G + C (%)	Contigs	N_50_ (bp)	Coverage (×)	PCGs	tRNAs	rRNAs	SRA ID	GenBank ID	BioSample ID
*S. putrefaciens*	DSM50426	4,352,486	44.3	32	510,942	61	4,017	96	14	SRR18741132	JALOCN000000000	SAMN27561646
*S. xiamenensis*	NZRM825	4,720,817	46.3	33	625,775	59	4,313	96	16	SRR19369596	JAMLGA000000000	SAMN28613652
*S. glacialipiscicola*	SM77	4,151,466	44.1	101	125,200	85	3,856	97	16	SRR18717573	JAKKSA000000000	SAMN25232827
*S. glacialipiscicola*	SM91	4,151,640	44.1	98	125,200	78	3,858	97	15	SRR18717572	JAKKSD000000000	SAMN25232830
*Shewanella* sp.	SW1	5,105,942	45.6	66	178,449	59	4,766	104	12	SRR18717588	JAKKSG000000000	SAMN25232833
*Shewanella* sp.	SW24	5,131,195	45.4	93	169,033	62	4,752	105	17	SRR18717587	JAKKSH000000000	SAMN25232834
*Shewanella* sp.	SW29	5,269,775	45.3	122	115,579	51	4,998	99	13	SRR18717576	JAKKSI000000000	SAMN25232835
*Shewanella* sp.	SW32	5,268,632	45.3	119	115,794	67	5,004	98	13	SRR18717565	JAKKSJ000000000	SAMN25232836
*Shewanella* sp.	SW36	5,107,263	45.6	68	178,402	55	4,757	104	12	SRR18717564	JAKKSK000000000	SAMN25232837
*Shewanella* sp.	SM20	5,058,412	45.3	153	73,372	58	4,820	103	16	SRR18717563	JAKKRK000000000	SAMN25232811
*Shewanella* sp.	SM21	4,893,123	45.3	43	301,835	62	4,490	98	16	SRR18717562	JAKKRL000000000	SAMN25232812
*Shewanella* sp.	SM23	5,050,234	45.3	72	226,807	55	4,639	104	17	SRR18717561	JAKKRM000000000	SAMN25232813
*Shewanella* sp.	SM29	5,030,501	45.2	110	134,150	70	4,684	99	11	SRR18717560	JAKKRN000000000	SAMN25232814
*Shewanella* sp.	SM32	5,192,630	45.3	193	66,739	57	4,983	95	16	SRR18717559	JAKKRO000000000	SAMN25232815
*Shewanella* sp.	SM34	5,224,009	45.1	135	107,555	50	4,989	103	17	SRR18717584	JAKKRP000000000	SAMN25232816
*Shewanella* sp.	SM35	5,226,293	45.1	139	107,555	54	5,007	104	13	SRR18717586	JAKKRQ000000000	SAMN25232817
*Shewanella* sp.	SM43	5,143,559	45.6	70	196,483	58	4,803	98	14	SRR18717585	JAKKRR000000000	SAMN25232818
*Shewanella* sp.	SM55	5,086,625	45.3	52	241,403	58	4,691	103	14	SRR18717583	JAKKRS000000000	SAMN25232819
*Shewanella* sp.	SM65	5,048,297	45.5	49	278,387	78	4,687	105	18	SRR18717580	JAKKRT000000000	SAMN25232820
*Shewanella* sp.	SM68	5,025,288	45.5	42	341,119	50	4,670	104	15	SRR18717582	JAKKRU000000000	SAMN25232821
*Shewanella* sp.	SM69	5,331,476	45.3	63	264,063	62	5,011	100	13	SRR18717581	JAKKRV000000000	SAMN25232822
*Shewanella* sp.	SM71	5,076,411	45.3	66	194,973	87	4,752	99	13	SRR18717579	JAKKRW000000000	SAMN25232823
*Shewanella* sp.	SM72	5,411,682	45.4	82	211,187	60	5,095	105	12	SRR18717578	JAKKRX000000000	SAMN25232824
*Shewanella* sp.	SM73	5,111,662	45.2	45	250,203	75	4,694	102	17	SRR18717577	JAKKRY000000000	SAMN25232825
*Shewanella* sp.	SM74	5,512,552	45.4	110	170,532	55	5,240	107	16	SRR18717575	JAKKRZ000000000	SAMN25232826
*Shewanella* sp.	SM78	5,333,096	45.1	142	95,342	61	5,121	105	14	SRR18717574	JAKKSB000000000	SAMN25232828
*Shewanella* sp.	SM87	5,234,487	45.3	105	144,165	55	4,950	97	18	SRR18717571	JAKKSC000000000	SAMN25232829
*Shewanella* sp.	SM95	5,232,876	45.4	76	169,717	59	4,889	99	15	SRR18717570	JAKKSE000000000	SAMN25232831
*Shewanella* sp.	SM96	5,242,714	45.1	90	172,535	64	4,952	97	12	SRR18717569	JAKKSF000000000	SAMN25232832
*Shewanella* sp.	SM101	5,290,430	45.3	153	105,230	62	5,055	112	19	SRR18717567	JAKKRH000000000	SAMN25232809
*Shewanella* sp.	SM102	5,075,919	45.3	66	173,102	63	4,741	96	12	SRR18717568	JAKKRI000000000	SAMN25232808
*Shewanella* sp.	SM103	5,277,435	45.2	121	97,874	45	5,028	104	15	SRR18717566	JAKKRJ000000000	SAMN25232810

**Figure 1:**
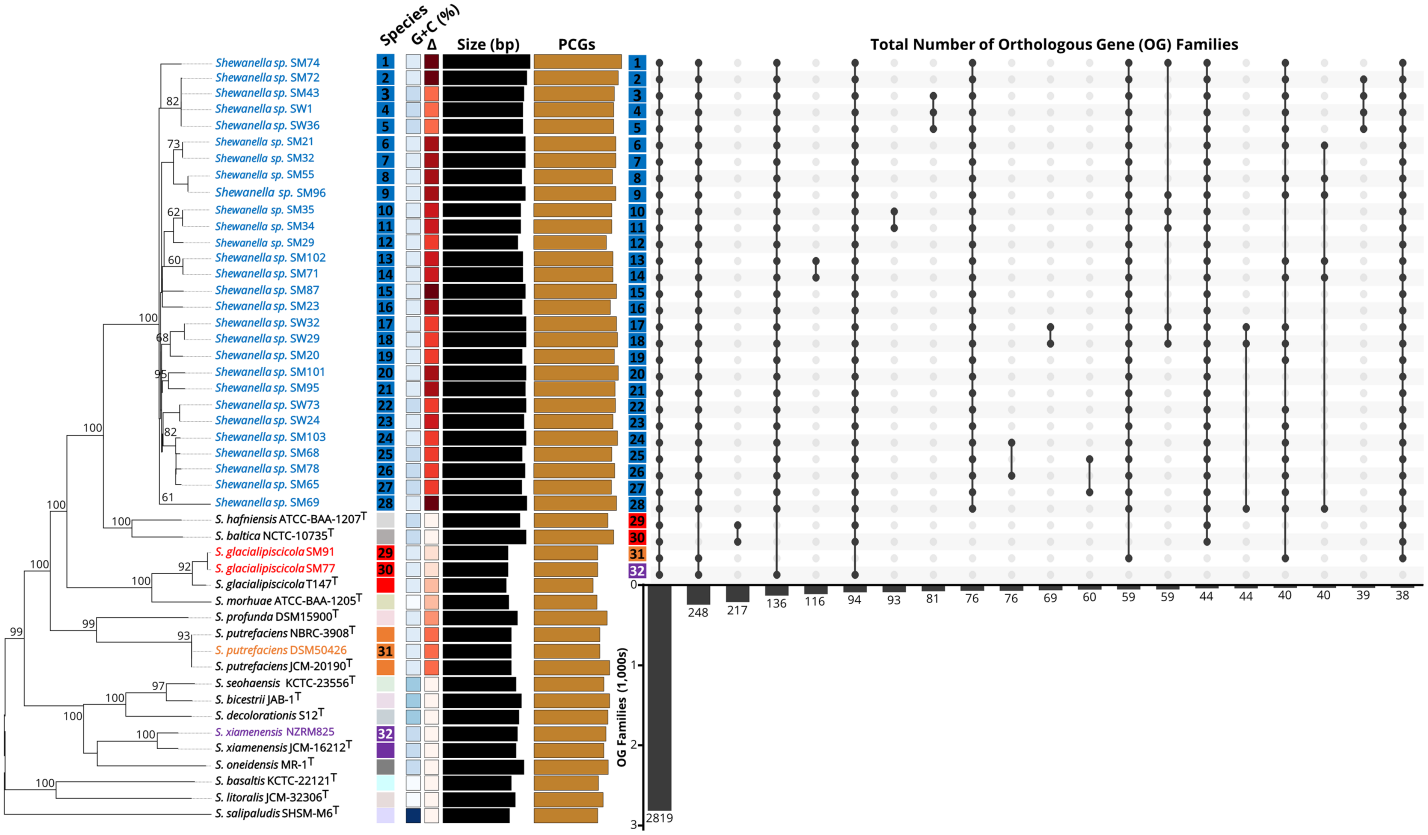
Phylogenomic species-tree reconstruction and homologous gene families of the *Shewanella* genomes. The phylogenomic tree (left) inferred with GBDP displays the pseudo-bootstrap support values from 100 replications above the branches. Type species are indicated with a superscripted T. The colored boxes to the right of each name refer to the species determined by dDDH cutoffs of 70 and 79%, respectively. The gradients further right indicate G + C content (blue) and Δ statistics (red) indicate overall low (light) to high (dark) accuracy of phylogenetic likeness. Black bars represent the approximate genome size and brown bars the protein-coding genes (PCGs). Genome names are colored to represent putative species grouping. The top 20 orthologous gene (OG) families across all 32 *Shewanella* proteomes is shown on the far right. Dark circles represent presence of OGs across genomes and total number of gene families shown in numbers below the bars.

At both the species and genus levels, the usefulness of complete or partial 16S rRNA gene sequence similarity as a taxonomic marker for *Shewanellaceae* has been limited (Martín-Rodríguez and Meier-Kolthoff, 2022). Thus, we investigated the taxonomy of our *Shewanella* genomes, using whole-genome sequence-based pairwise methods including TETRA correlation, average nucleotide identity based on BLAST+ (ANIb), and genome-wide identity indexes using digital DNA:DNA hybridization (dDDH) analyses. Our analyses resulted in the identification of 28 *Shewanella* spp. strains that represent uncharacterized clades or novel species (with TETRA/ANIb of >0.999/>96.6%), including six putative subspecies that await formal description (Figure 1). From a taxonomic perspective, our phylogenomic reconstruction supported the reassignment of both *Shewanella* sp. SM77 and SM91 to *Shewanella glacialipiscicola* due to high sequence similarity (with TETRA/ANIb of ~0.975/>85.0% and dDDH of >90% for both) to the *S. glacialipiscicola* T147^T^ type strain. The exceptions were *S. putrefaciens* DSM50426, and *S. xiamenensis* NZRM825 with a high G + C content of 46.3% and as determined by dDDH and ANIb that will require further experimental investigation. Our genome-wide analyses have highlighted the complexity of the taxonomic relationships within the genus *Shewanella* with all *Shewanella* type strains currently sequenced and publicly available from NCBI/TYGS (Martín-Rodríguez and Meier-Kolthoff, 2022). The reported *Shewanella* spp. genome collection may serve as a valuable resource for prospective work investigating their taxonomic diversity and reference for unravelling their genetic mechanisms associated with spoilage of vacuum packaged meat.

Genome-wide replication events in *Shewanella* have been considered as a mechanism for diversification and adaptation to the environment. To analyze the diversity and evolutionary conservation of the 32 *Shewanella* genomes, we compared the protein sets for each genome in a pairwise manner to identify sequence similarities and orthologous genes between the different species. Our comparative analysis of the expansions and contractions of gene families revealed markedly different orthology profiles and models of gene gain or loss, but particularly for the major *Shewanella* cluster (Figure 1). In total, we identified 2,819 orthogroups with all species present, 2,683 single-copy conserved orthologs, 836 one-to-many, and 434 many-to-many co-orthologous genes, with 50 species-specific orthogroups consisting of 106 species-specific genes among the 32 *Shewanella* genomes, indicating that these genes have expanded to varying degrees. While the comparative analysis of the *Shewanella* gene families will be helpful to identify the retention and loss of key gene family members after the differentiation of various species, further phenotypic characterization will be required to routinely discriminate between closely related species that might exist within any given meat sample.

### Spoilage and adaptation to stress genes

**Figure 2:**
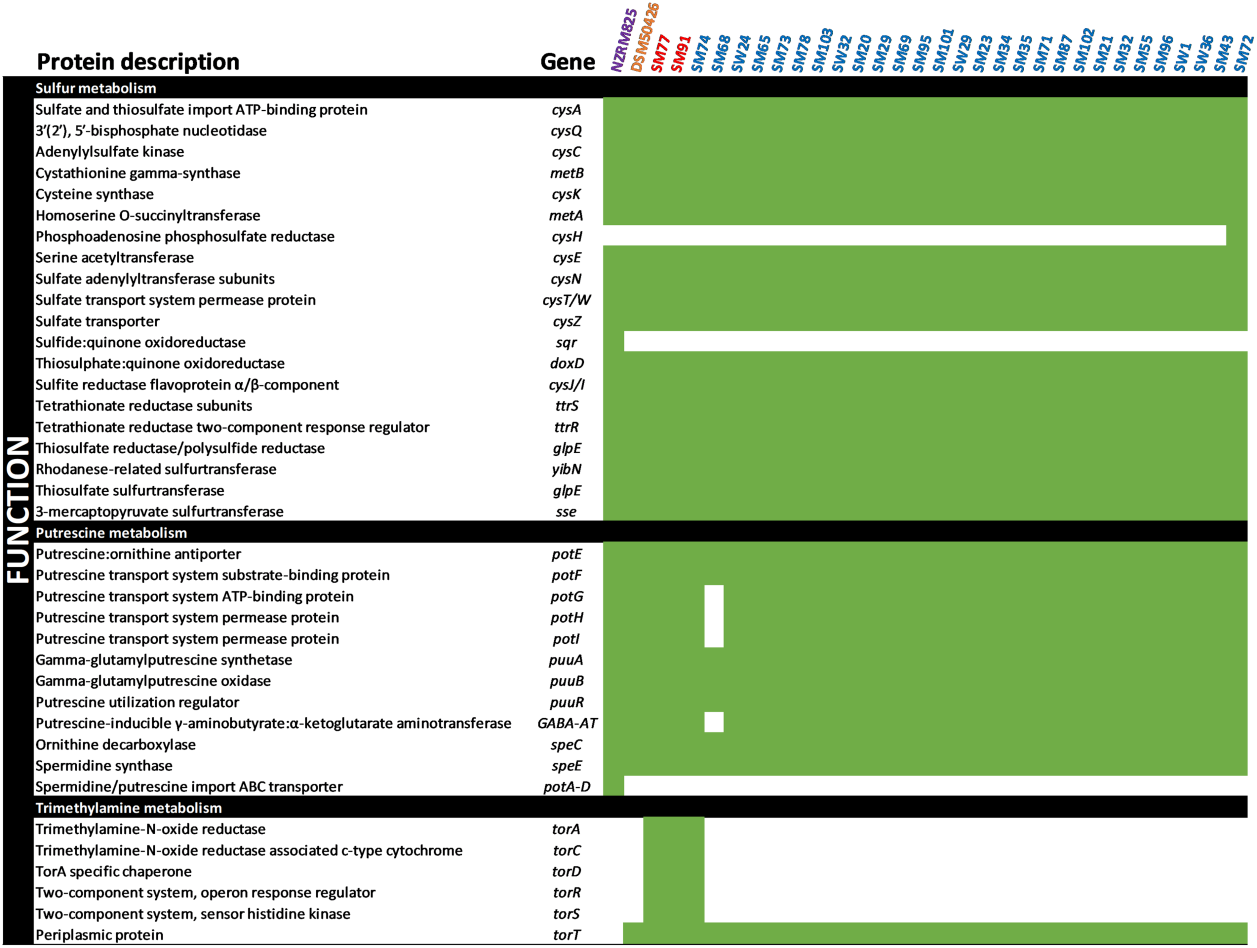
Occurrence of spoilage-related genes in *Shewanella* genomes. Presence (green) or absence (white) for each gene is depicted.

**Figure 3:**
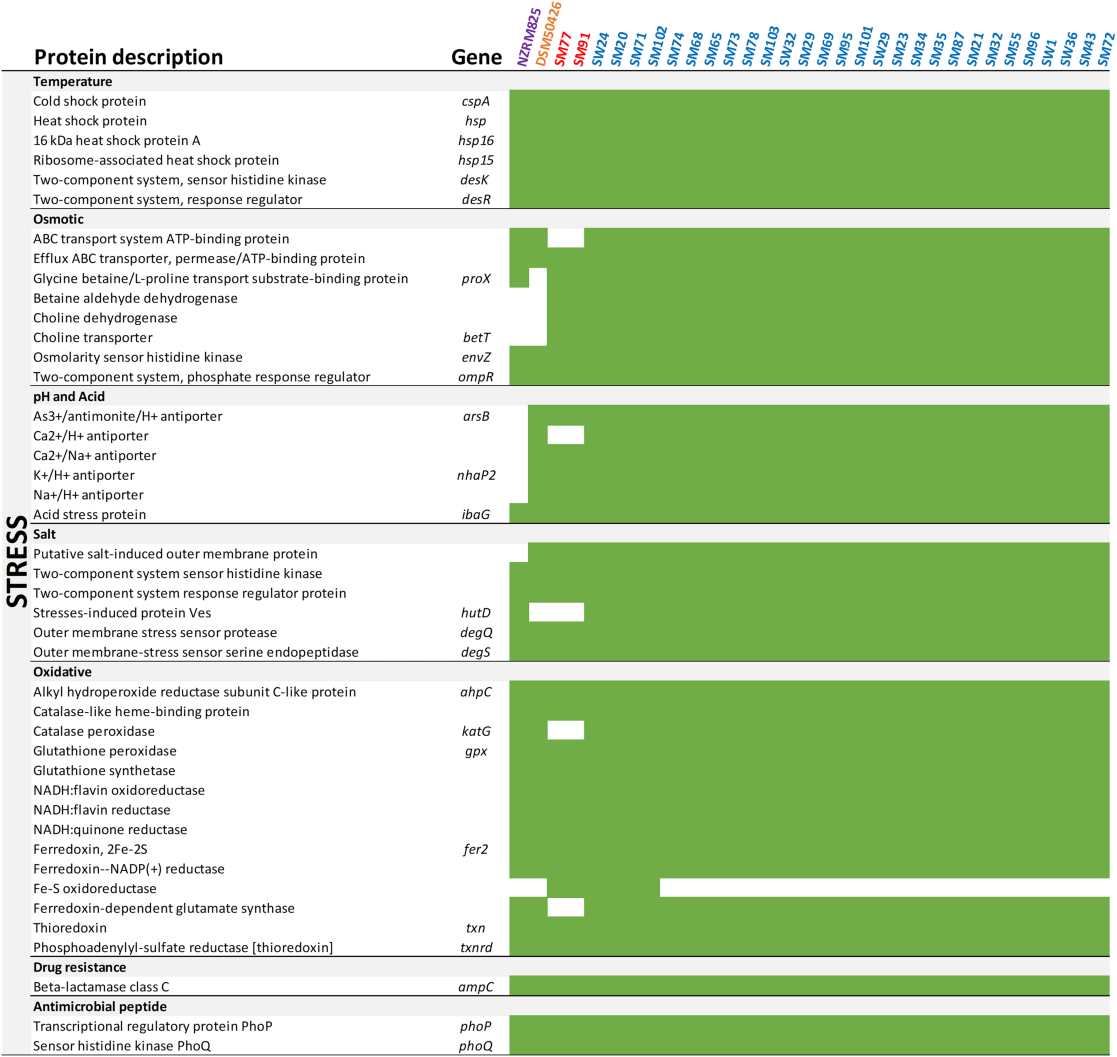
Presence of stress-related genes of *Shewanella*. Presence (green) or absence (white) for each gene is depicted.

The spoilage potential of *Shewanella* spp. has been associated with sulfur, putrescine, and trimethylamine metabolism (Remenant et al., 2015). To better understand the spoilage potential of *Shewanella* spp. strains in the spoiled vacuum-packaged refrigerated lamb, genes were annotated against the KEGG database using the EGGNOG mapper of the Blast2GO suite (Figures 2, 3).

The specific bacterial spoilage of vacuum-packaged meat products has been associated with sulfur metabolism, and the production of hydrogen sulfide gas (H_2_S) responsible for putrid off-odors in such products has been associated with the presence of *Shewanella putrefaciens* (Borch et al., 1996). As shown in Figure 2, a series of the *cys* genes involved in sulfur metabolism and responsible for H_2_S production were identified in the *Shewanella* genomes. Genes encoding sulfate adenylyltransferase (*cysN*, EC 2.7.7.4), adenylylsulfate kinase (*cysC*, EC 2.7.1.25), and sulfite reductase (*cysJ*/I EC 1.8.1.2) were found, suggesting that the *Shewanella* spp. strains have good potential for sulfur metabolism. However, only *Shewanella* spp. strain SM72 was found to encode a phosphoadenosine phosphosulfate reductase (*cysH*, EC 1.8.4.8) that reduces 3′-phosphoadenosine-5′-phosphosulfate (PAPS) to sulfite. Notably, our genome analysis identified genes encoding tetrathionate reductase (*ttrS*, EC 1.8.5.5) and cysteine synthase B (*cysK*, EC 2.5.1.47), suggesting that tetrathionate may also be preferentially reduced to sulfide by all *Shewanella* spp. strains.

Biogenic amines such as putrescine and spermidine are produced *via* decarboxylation of various amino acids and their transport systems have also been described (Remenant et al., 2015). A series of *pot* genes encoding putrescine transport systems and permease proteins (*potE-I*), as well as the putrescine:ornithine antiporter (*potE*) were found in all *Shewanella* genomes, except *Shewanella* spp. strain SM68 that lacked *potG-I*. In addition, genes encoding *L*-ornithine decarboxylase (*speC*, EC4.1.1.17) and spermidine synthase (*speE*, 2.5.1.16) were also found in all *Shewanella* genomes. The occurrence of these key enzymes indicates that the reported *Shewanella* spp. strains could produce putrescine from *L*-ornithine and spermidine.

Recently, the genomic potential of *Shewanella* species for their ability to decompose trimethyl-amine-N-oxide (TMAO) into trimethylamine (TMA) resulting in the production of a strong odor has been elucidated (Li et al., 2020). The full complement of genes required for de-oxygenation of TMAO into TMA and subsequent metabolism, including trimethylamine-N-oxide reductase (*torA*), TorA specific chaperone (*torD*), sensor histidine kinase (*torS*), periplasmic protein TorT, were found in only *Shewanella* spp. strains SM74, SM77, and SM91. Our findings provide evidence that the regulatory genes of trimethylamine metabolism may be highly conserved across *Shewanella* species associated with spoilage of a variety of meat products and warrant further investigation.

Ultimately, the success of spoilage microorganisms is dependent on their ability to survive under some critical environmental stresses associated with the storage and processing of food. Figure 3 summarizes the occurrence of key stress-related genes that regulate the adaptability of *Shewanella* spp. to temperature, osmotic, salt, oxidative, antimicrobial peptide, and drug resistance stresses. The presence of genes encoding cold shock protein (*cspA*), sensor histidine kinase (*desK*), and response regulator (*desR*) may allow *Shewanella* spp. to perform transcription during prolonged cold exposure and improve their tolerance to low temperatures. Also, *Shewanella* spp. all contain genes encoding ABC-type transport system permease and transport proteins, osmolarity sensor histidine kinase (*envZ*), and phosphate regulon response regulator (*ompR*) related to osmotic pressure. Furthermore, genes encoding sodium-proton antiporters (Na^+^/H^+^) responsible for the maintenance of homeostasis and salt tolerance were abundant across all strains. For example, *degS* functions as the principal regulator of degradative enzymes and encodes the outer membrane-stress sensor serine endopeptidase. Lastly, *ampC* that encodes β-lactamase class C involved in drug resistance and *phoP/Q* associated with antimicrobial peptide resistance have been identified in all *Shewanella* spp. strains. Overall, the *Shewanella* spp. genome sequences are a valuable resource for future investigations aiming to develop new detection and prevention strategies targeting food spoilage bacteria.

## Data availability statement

The genome sequence and associated data for Shewanella strains have been deposited under the GenBank BioProject accession number PRJNA574489. Detailed information can be found in Table 1 regarding BioSample and Sequence Read Archive (SRA) accession numbers: https://www.ncbi.nlm.nih.gov/bioproject/?term=PRJNA574489.

## Author contributions

GB and JM conceived the study and acquired the funding. FP and AG collected the samples and conducted the experiments. NP performed the bioinformatics analysis, collated the data, and wrote the manuscript with input from all other authors. All authors contributed to the article and approved the submitted version.

## Funding

This research was supported by the AgResearch Ltd. Strategic Science Investment Fund (grant PRJ0331443).

## Conflict of interest

NP, FP, AG, GB, and JM were employed by AgResearch Ltd.

## Publisher’s note

All claims expressed in this article are solely those of the authors and do not necessarily represent those of their affiliated organizations, or those of the publisher, the editors and the reviewers. Any product that may be evaluated in this article, or claim that may be made by its manufacturer, is not guaranteed or endorsed by the publisher.

## Footnotes

^1^ https://ggdc.dsmz.de

^2^https://www.R-project.org/ (Accessed September 2, 2022).
